# Contraceptive complication: Emergence of a bladder stone - A case report

**DOI:** 10.1016/j.ijscr.2024.109622

**Published:** 2024-04-06

**Authors:** Wael Gazzah, Zied Mansi, Bacem Zaidi

**Affiliations:** a University of Sousse, Department of Urology, Ibn El Jazzar Hospital, Kairouan, Tunisia; b University of Sousse, Department of Orthopedic Surgery, Ibn El Jazzar Hospital, Kairouan, Tunisia; c University of Sousse, Department of Surgery, Ibn El Jazzar Hospital, Kairouan, Tunisia

**Keywords:** Urinary bladder calculi, Intrauterine devices, Uterine perforation, Urolithiasis, Foreign-body migration

## Abstract

**Introduction and importance:**

Bladder stones, although rare in a healthy bladder, can emerge due to various factors, including obstructions in urinary flow, recurrent infections, and foreign bodies. Intrauterine contraceptive devices (IUCDs) are known for their potential to migrate from the uterine cavity, leading to unusual complications such as bladder stone formation.

**Case presentation:**

A 52-year-old woman, previously treated for a complicated urinary tract infection, presented with intermittent lower abdominal pain, dysuria, and hematuria. She had a history of an IUCD insertion 15 years earlier, which was later documented as missing. Diagnostic imaging revealed a large bladder stone, encasing the previously inserted IUCD. An open vesicolithotomy was performed, during which a stone measuring 6 × 5 cm was removed, revealing the IUCD within. The patient had an uncomplicated recovery with no further urinary tract infections at a 6-month follow-up.

**Clinical discussion:**

The migration of an IUCD can lead to various complications, depending on its final location. The formation of bladder stones around a migrated IUCD is a rare but significant complication, necessitating a thorough diagnostic approach. Radiography and ultrasonography proved sufficient for diagnosing the intravesical migration in this case.

**Conclusion:**

This case underscores the importance of considering a migrated IUCD in the differential diagnosis of patients presenting with urinary symptoms, especially those with a history of a missing IUCD. Timely diagnosis and management are crucial in preventing further complications.

## Highlights

• Rare bladder stone from a migrated IUD, diagnosed after 15 years.
• Presented with pain, dysuria, hematuria and recurrent UTIs.
• X-ray and ultrasound confirmed large bladder stone encompassing the contraceptive device.
• Open vesicolithotomy successfully removed the bladder stone and the intrauterine device, patient recovered well.

## 1 Introduction

Bladder stones, although rare in a normally functioning bladder, can develop due to various causes, such as obstructions of the urinary outflow, ongoing or repeated urinary infections, and the presence of foreign objects in the bladder [1]. A less common iatrogenic source of bladder stones is the displacement of an intrauterine contraceptive device (IUCD) [2]. IUCDs carry a known risk of uterine perforation and subsequent migration outside the uterus. Studies report that uterine perforations occur at a frequency of approximately 1.2 to 1.6 per 1000 IUCD insertions [2]. Predominant locations of a displaced IUCD include the omentum, rectum, sigmoid colon, peritoneum, and, in particular, the bladder [3]. The specific symptoms resulting from IUCD migration vary depending on the final location of the device. When an IUCD migrates to the bladder, it usually results in lower urinary tract symptoms, and these can manifest even in the absence of bladder stones.

This case report delineates a notably atypical instance of IUCD-induced bladder lithiasis, distinguished by the considerable size of the stone and a prolonged asymptomatic interval prior to detection. This adds a novel perspective to the spectrum of IUCD-related complications. Initially considered lost, this IUCD led to the formation of a sizable secondary bladder stone, which was only detected 15 years after insertion. In particular, the prolonged period without symptoms in this case contributed to the significant growth of the bladder stone and subsequently delayed medical consultation and care.

## 2 Case presentation

A 52-year-old woman patient presented to our Urology Department for the treatment of a significant bladder stone, after settling a complex urinary tract infection (UTI) involving the upper tract. During the preceding six months, she experienced intermittent lower abdominal pain, dysuria, and hematuria. During this period, she suffered three episodes of simple urinary tract infections, managed by her primary care physician. Each episode involved the isolation of *Proteus mirabilis* from urine cultures, with treatments guided by antibacterial sensitivity, mainly a week-long regimen of coamoxiclav. Following the third UTI, nitrofurantoin was prescribed as a prophylactic urinary antiseptic.

**Fig. 1:**
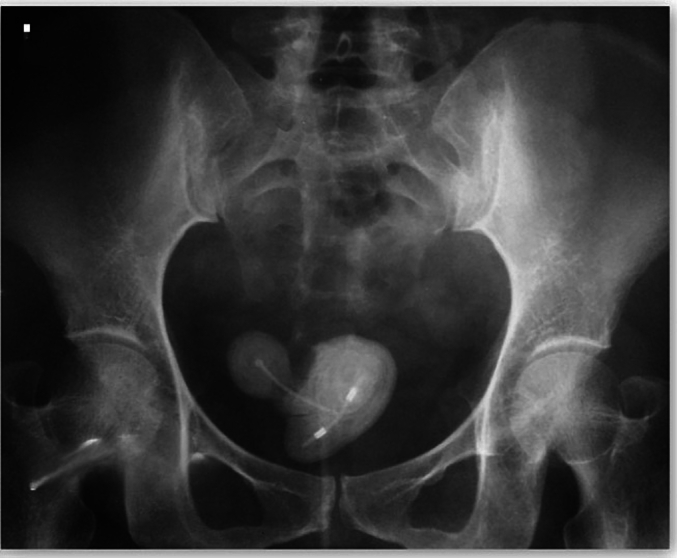
plain X-ray of the pelvis; a calcified bladder mass with the embedded intrauterine contraceptive device is observed.

**Fig. 2:**
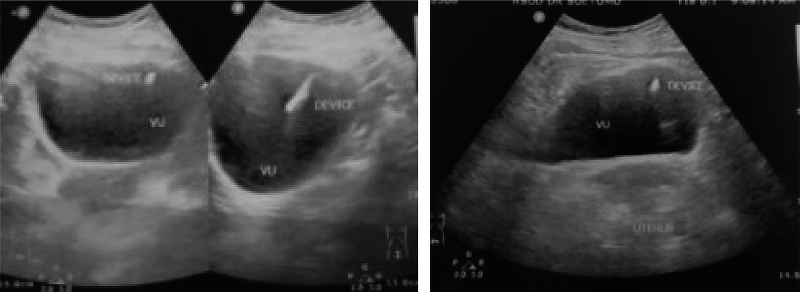
Ultrasound view of the migrated IUCD in the bladder.

On presentation of her fourth more severe UTI, the patient underwent a comprehensive diagnostic evaluation. The clinical evaluation revealed septic signs, including tachycardia, moderately elevated blood pressure, pronounced fever, and increased respiratory rate. Laboratory investigations showed significant leukocytosis, although liver and renal functions were within normal parameters. Radiographic imaging of the kidneys, ureters, and bladder, along with ultrasound examination, revealed a conspicuously large bladder stone, exhibiting three protruding formations and encapsulating a typical copper intrauterine contraceptive device (IUCD) (Fig. 1, Fig. 2). Additionally, ultrasound identified left-sided pyelonephritis. A diverse microbial spectrum, including *coliforms* and *Proteus* species, was identified in urine cultures. The patient received intravenous cefotaxime based on antibacterial sensitivities for one week, and the treatment was supplemented with nitrofurantoin in anticipation of surgical intervention.

Further history analysis revealed that the patient had an IUCD placed 15 years ago, following the birth of her third child. In particular, the IUCD was documented as missing two years after insertion when routine examination did not locate the threads and the patient did not pursue subsequent follow-up. The recollection of the IUCD's existence was prompted only upon direct inquiry. With no urinary or abdominal symptoms reported in the recent past and an unremarkable medical history, her risk factors for stone formation appeared minimal. Physical examinations, both general and abdominal, did not reveal additional abnormalities.

**Fig. 3:**
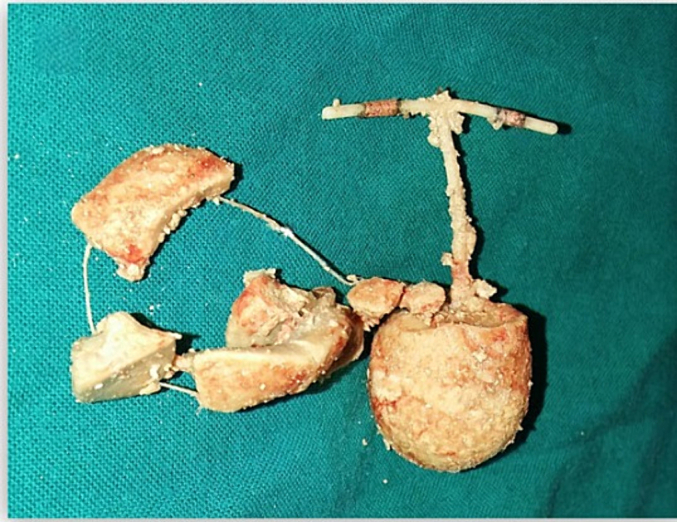
photograph of the bladder stone removed from the bladder.

A diagnosis was established for a bladder stone that encapsulates a migrated IUCD. The substantial size of the stone required an open vesicolithotomy, scheduled four weeks later. Intraoperatively, the IUCD was discovered entirely encased within the bladder stone, rather than partially embedded in the bladder wall, underlining the chronicity of migration and the progressive formation of stones. A large bladder stone, measuring 6 × 5 cm and featuring three extensions that mimicked the shape of the IUCD, was extracted. The stone was sectioned, revealing the IUCD enveloped within. The threads of the IUCD were also entirely encased in stone material (Fig. 3).

The postoperative course was uneventful, with the patient remaining asymptomatic after extraction. Follow-up at six months did not show recurrence of UTI or related complications.

## 3 Discussion

The transmigration of intrauterine contraceptive devices (IUCD) usually originates from an initial traumatic perforation of the uterus or a prolonged inflammatory process. However, the exact mechanisms behind these occurrences are not fully understood. Copper-based IUCDs, renowned for their contraceptive effectiveness, induce an inflammatory response that serves the dual purpose of contraception and, potentially, contributes to chronic uterine perforation and device migration [4].

Migration of IUCDs resulting in bladder stone formation, while documented, presents different challenges and insights in each case. A comparative review of similar incidences, such as those documented by Akhtar et al. [2], Johri and Vyas [3], and Aggarwal et al. [5], reveals a spectrum of presentations and complications, ranging from asymptomatic migrations to severe cases characterized by urinary tract infections and substantial stone development. The case we report here is marked by an extended asymptomatic period after migration of IUCD, culminating in the development of a significantly large bladder stone, undiagnosed for a prolonged period. This case underscores the unpredictability of IUCD migrations and their capacity for dormant, yet significant complications.

Unlike numerous reports in which complications appear shortly after IUCD insertion, the case in question is distinguished by a notable 15-year lapse between device insertion and the onset of symptoms, alongside the formation of a large bladder stone. This deviation from typical IUCD migration timelines and the presentation of symptoms offer crucial information on the management and understanding of these complications in both the urological and gynecological domains.

In this particular case, the patient initially noticed the IUCD threads, but they were deemed missing two years after insertion, a situation exacerbated by her lack of adherence to recommended follow-up appointments. The presence of a foreign body such as an IUCD in the bladder, or even its partial encroachment on the bladder wall, can instigate a range of lower urinary tract symptoms. Interestingly, in this case, the patient did not show symptoms for 13 years after the presumed migration of the IUCD, and the eventual manifestation of symptoms was exclusively attributable to the considerable bladder stone.

This case illustrates a chronic, asymptomatic migration of an IUCD to the bladder, which remained undetected until the emergence of symptoms induced by a secondary bladder stone. The distinct impression of the IUCD on the stone, coupled with the concentric layering of the stone, as observed in our patient's radiograph, are indicative of a secondary bladder stone forming around a migrated IUCD. This presentation pattern, parallel to the case reported by Aggarwal et al. [5], demonstrates the diagnostic utility of radiographs and ultrasound in identifying intravesical migration of IUCD [5,6]. However, it should be noted that in cases of migration of IUCD to other anatomical sites, computed tomography (CT) scanning may be crucial for precise localization [7].

The nature and severity of complications from IUCD migration are largely dependent on the final location of the device. Both intraperitoneal and extraperitoneal migration paths have been documented. The omentum, for example, is a common site for intraperitoneal migrations, with various complications associated with these cases. Robayo-Amortegui et al. described a rare case of sigmoid colocolic fistula resulting from intraperitoneal IUCD [8]. On the contrary, the bladder, rectum, and ureter are recognized sites of extraperitoneal migration. Numerous reports document intravesical migrations, often culminating in bladder stone formation (vesicolithiasis) [2,5,[9], [10], [11]], with documented cases of rectal perforation [12] and ureteral erosion [13] due to migrated IUCD.

In the situation of our patient, the multifaceted origin of her bladder symptoms unraveled only after comprehensive imaging and detailed exploration of her medical history. In particular, bladder symptoms can manifest from even a partial invasion of the bladder wall by an IUCD, without complete transmigration [4]. Therefore, a high index of suspicion is warranted in clinical evaluations of patients with bladder symptoms and a history of intact or missing IUCDs. This case further highlights the imperative for meticulous investigations in patients with unaccounted for IUCDs.

The decision to proceed with an open vesicolithotomy, rather than cystoscopic removal, was influenced by several factors. Firstly, the substantial dimensions of the stone, which measures 6 × 5 cm, along with its intricate morphology that features multiple protrusions, cast doubts on the feasibility and safety of a cystoscopic approach. The likelihood of incomplete stone removal and the potential for bladder trauma were considered significant risks. The open surgical technique facilitated a thorough and secure extraction of both the stone and embedded IUCD, simultaneously mitigating the risk of bladder damage. This scenario accentuates the need to customize the surgical strategy to accommodate the specificities of each case, especially when faced with unusually large or intricately formed bladder stones.

Considering the erratic nature of IUCD migrations, the prevailing recommendation advocates the extraction of any migrated IUCD once its exact location has been determined. Such a proactive measure is intended to avert potential adverse outcomes stemming from the device's continued residence in an unintended anatomical location.

This case serves to reinforce the need for increased vigilance in the treatment of patients with a history of IUCD use, particularly in instances where they present with urinary symptoms. Medical practitioners should always consider the possibility of IUCD migration in their differential diagnoses, regardless of the time since insertion. Ensuring regular follow-up and timely comprehensive diagnostic evaluations, including imaging, is critical in patients with missing IUCD threads to avoid the risk of late identification of potential complications. In cases where bladder stones are complicated by the presence of IUCD, a meticulous evaluation is crucial to determine the most suitable surgical intervention. This requires judiciously weighing the risks associated with invasive procedures against the benefits of complete removal and alleviation of symptoms.

## 4 Conclusions

A displaced intrauterine contraceptive device (IUCD) can serve as the focal point for the development of secondary bladder stones. It is imperative to maintain a higher level of suspicion in cases involving patients with unaccounted for IUCDs who present with bladder-related symptoms. The intricately detailed imprint of the IUCD encased within the bladder stone, along with the stratified composition of the stone material, are hallmark features indicative of a secondary bladder stone formed around an IUCD. To diagnose cases of migration of IUCD to the bladder, radiographic imaging and ultrasound have been proven to be sufficient and effective diagnostic tools.

## Patient perspective

The patient expressed relief and satisfaction with the treatments received, highlighting their effectiveness and the care provided by the medical team.

## Informed consent

Written informed consent was obtained from the patient for the publication of this case report and any accompanying images. A copy of the written consent form is available for review by the Editor-in-Chief of this journal upon request.

## SCARE guidelines

This study was reported in line with the SCARE criteria [14].

## Ethical approval

This study is exempt from ethical approval as per the policies of Ibn El Jazzar Hospital.

## Funding

No funding was received for conducting this study.

## Author contribution

All authors have contributed equally to the work reported in this manuscript, including the conception, design, execution, data acquisition, analysis and interpretation, and the drafting and revising of the manuscript for important intellectual content.

## Guarantor

Wael Gazzah.

## Research registration number

SRN 00234/2024.

## Conflict of interest statement

The authors declare that they have no conflicts of interest concerning this article.
